# Cat Scratch Disease Is an Entity Often Diagnosed in Breast Imaging Department During Axillary Lymph Node Assessment

**DOI:** 10.7759/cureus.9272

**Published:** 2020-07-19

**Authors:** Michael V Lin, Nga T Nguyen, You-Wen Qian, Vincent T Phan, Quan D Nguyen

**Affiliations:** 1 Radiology, University of Texas Medical Branch, Galveston, USA; 2 Pathology, University of Texas Medical Branch, Galveston, USA

**Keywords:** cat scratch disease, bartonella henselae, breast cancer, breast mass, axillary lymphadenopathy

## Abstract

Cat scratch disease (CSD) is an infectious disease process of generally immunocompetent children and young adults. This infection can be introduced through skin trauma by direct exposure to the saliva of an infected kitten or cat. CSD is typically associated with constitutional symptoms and self-limited regional lymphadenopathy. In the sole presence of swollen lymph nodes, however, the differential diagnosis for CSD is relatively broad, including an active infection, an ongoing inflammatory process, and a metastatic process. CSD can present as axillary lymphadenopathy without typical constitutional symptoms. With proper clinical and laboratory investigation, CSD can be accurately identified and correctly diagnosed, as demonstrated in this case series featuring five symptomatic young adults with axillary lymphadenopathy. Breast imaging clinic specializes in lymph node assessment because metastatic lymphadenopathy is one of the most common presenting signs of breast cancer. Most isolated axillary lymphadenopathy without breast mass is benign reactive lymphadenopathy, but biopsy is necessary to exclude malignancies, such as metastatic lymphadenopathy or lymphoma.

## Introduction

The presence of unexplained swollen lymph nodes may be the only clinical finding in several diseases. Axillary lymph node, which drains the arm, breast, and thoracic wall, when palpable can indicate both benign and malignant processes, including infections, lymphoma, breast cancer, silicone implants, and melanoma. Palpable axillary nodes are more often related to benign rather than malignant disorders [1]. However, when cancer is identified, the most common tumor causing axillary lymphadenopathy is breast cancer. The incidence of breast cancer in both male and female patients with metastatic axillary adenopathy is 50% or higher [2]. Therefore, axillary adenopathy, although mostly benign, often requires further investigation with imaging or possible node biopsies.

Cat scratch disease (CSD) is a zoonotic disease caused by gram-negative bacillus Bartonella henselae (B. henselae) with the highest incidence of 6.4 cases per 100,000 population in the southern United States [3]. In an immunocompetent individual, CSD starts as a localized skin lesion over the course of three to ten days after inoculation by B. henselae from direct exposure to cat scratch, cat bite, or cat fleas. In terms of evolution, the skin lesion progresses through vesicular, erythematous, papular, pustular, or nodular stages with the persistence of skin lesions for one to three weeks. Within approximately two weeks of cutaneous inoculation, regional lymphadenopathy or lymphadenitis typically becomes evident as tenderness and erythema ensue proximal to the primary infection site. Constitutional symptoms, such as fever, headache, and malaise, often develop over the course of one to two weeks after inoculation [4]. The location for regional lymphadenopathy is variable, dependent on the inoculation site, and most frequently involves the anterior cervical, preauricular, supraclavicular, epitrochlear, axillary, inguinal, and femoral lymph nodes [5]. Atypical manifestations of CSD include visceral organ involvement, rare Parinaud’s oculoglandular syndrome, encephalitis, osteomyelitis, and endocarditis. Immunocompromised or immunosuppressed patients may also be susceptible to developing bacillary peliosis and bacillary angiomatosis.

Most cases of CSD are consistent with a clinical picture of an infectious process, including a history of exposure to cats, identification of inoculation site by cat scratch of insect bites, lymphadenitis, positive skin-test reaction, negative lab investigation for other causes of lymphadenopathy, and characteristic histopathologic findings of a biopsied lymph node [6]. However, atypical CSD can present without classic constitutional symptoms and only with the presence of axillary lymphadenopathy. Risk factors for malignancy include advancing age, male sex, white race, supraclavicular location of the nodes, and presence of systemic symptoms such as fever, night sweats, and unexplained weight loss, which sometimes overlap with atypical presentations of CSD. In the five cases below, axillary lymphadenopathy was present in all patients, two of whom had atypical clinical manifestations with isolated axillary lymphadenopathy and without constitutional symptoms.

## Case presentation

Case 1

**Figure 1:**
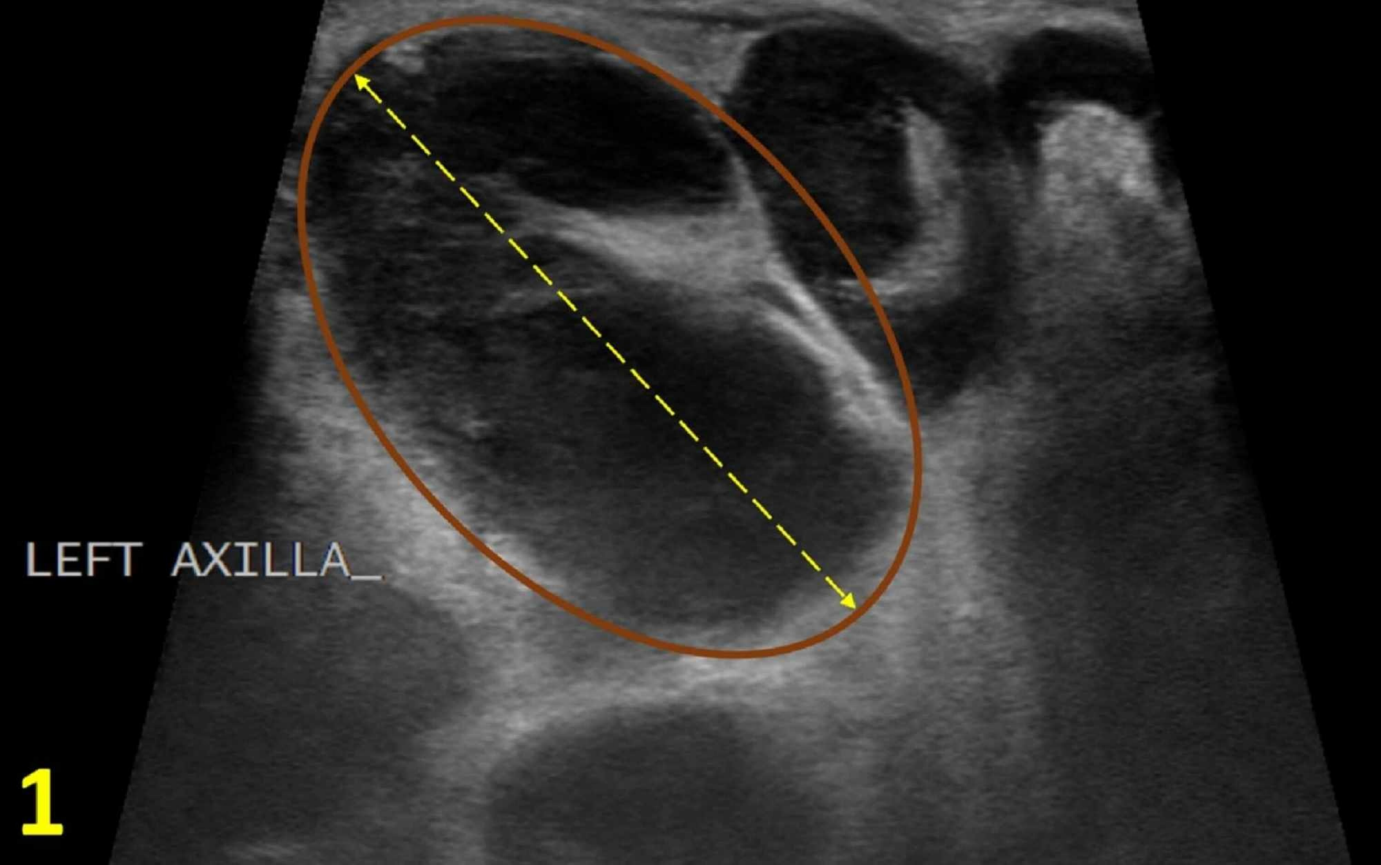
Left Axilla Diagnostic Ultrasound Enlarged axillary lymph node with cortical thickening (brown oval) measuring up to 33 mm (yellow double arrow).

An 18-year-old male patient presented with a new, large, and tender lump under his left axilla for two weeks with associated chills, night sweats, and left thumb lesion for one week. The patient reported sleeping with his cat and experiencing a recent cat bite. His family history was significant for his grandmother with an unknown type of cancer in the axillary region. On physical examination, two tender subcutaneous nodules in the left axilla, measuring 5 cm and 1 cm in diameter, respectively, were discovered. On the left thumb, an erythematous and excoriated papule was noted. Given his clinical presentation, social history, family history, and physical examination, the differential diagnosis included lymphoma, reactive lymphadenopathy, abscess, deep epidermal inclusion cyst, and CSD. An ultrasound revealed enlarged left axillary lymph nodes with cortical thickening, measuring up to 33 mm, prompting further investigation with axillary lymph node biopsy (Figure 1). On tissue examination of the biopsy, necrotizing lymphadenitis was identified, which is consistent with CSD. To confirm the diagnosis, serology for B. henselae was subsequently ordered resulting in an immunoglobulin G (IgG) titer against B. henselae of 1:256 and an immunoglobulin M (IgM) titer of >1:256. No monoclonal B-cell or abnormal T-cell populations were detected on flow cytometry.

Case 2

**Figure 2:**
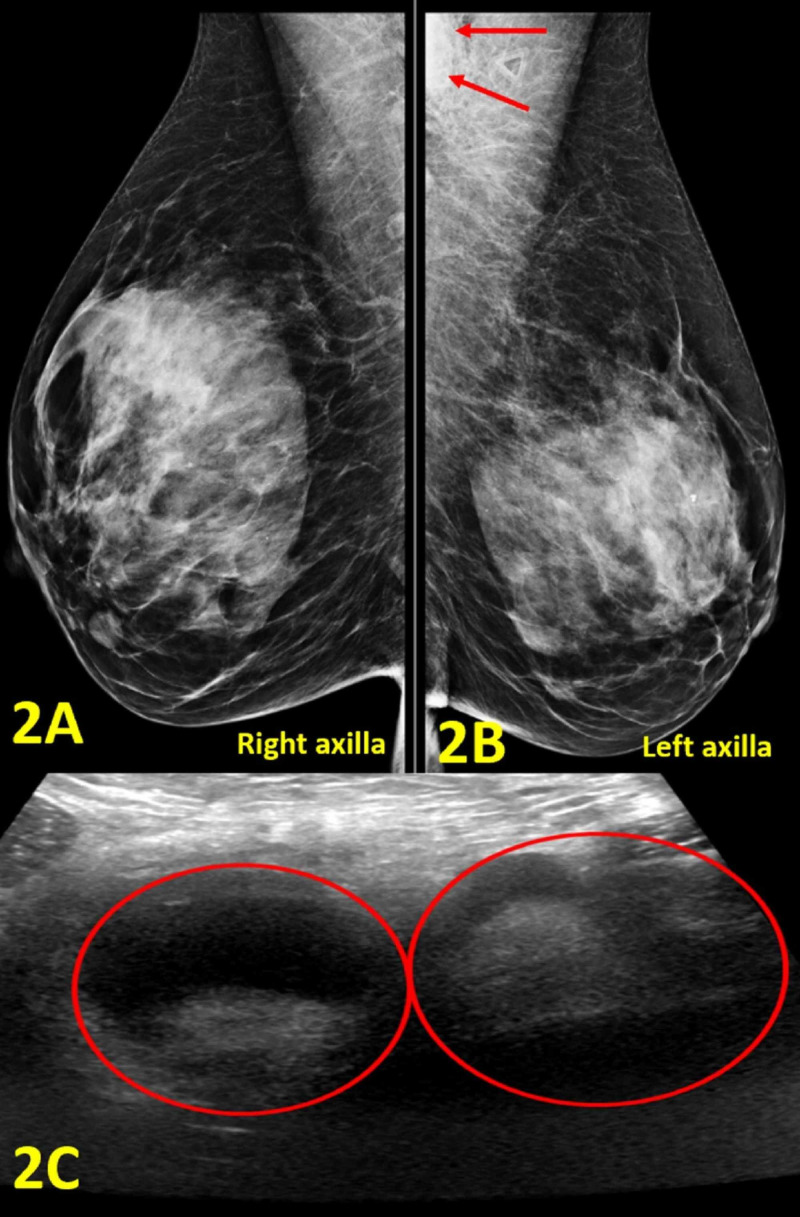
Imaging of Bilateral Breasts and Axillae (A) Right axilla diagnostic mammogram demonstrates no abnormal findings. (B) Left axilla diagnostic mammogram shows an enlarged lymph node in the left axilla (outlined by red arrows). (C) Left axilla diagnostic ultrasound shows two adjacent enlarged axillary lymph nodes with cortical thickening measuring 31 mm and 27 mm in diameter, respectively (red circles).

A 47-year-old woman presented with a painful, compressible, and mobile lump in the left axilla for three days without discharge or erythema. Prior to lump presentation, the patient had intermittent elbow and arm pain, lasting approximately 45 minutes. She denied fevers, chills, and night sweats. Her family history was significant for a father with liver cancer and maternal grandmother with bone cancer. She worked as a veterinarian and was a former smoker of five pack-years. On physical examination, there was a compressible, mobile, tender lump measuring 30 x 20 mm in the left axilla. Mammogram revealed normal right breast and left axillary lymphadenopathy (Figures 2A, 2B). The ultrasound showed two adjacent enlarged axillary lymph nodes measuring 31 mm and 27 mm in diameter, respectively (Figure 2C).

**Figure 3:**
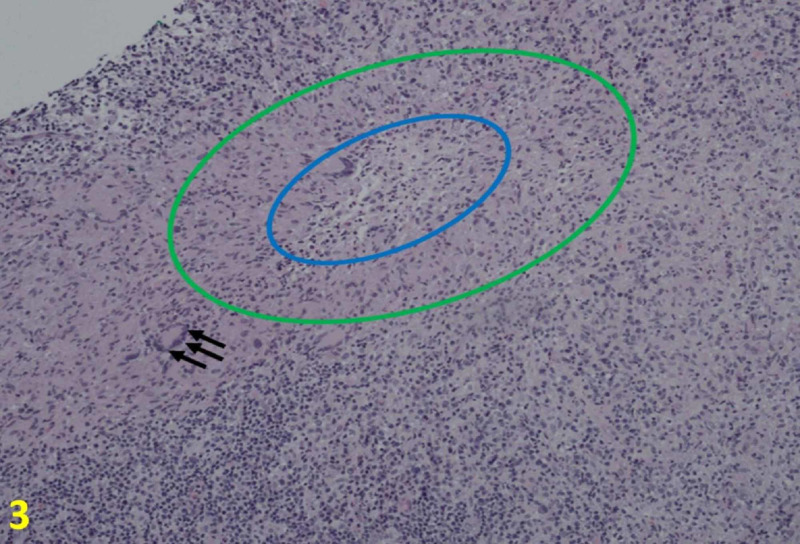
Histologic Findings of Cat Scratch Disease at 10× Core needle biopsies show necrotizing lymphadenitis that partially effaces the lymph node architecture. In the necrotic area, there are microabscess (blue oval) with neutrophils and surrounding histiocytes, some of which are palisading (areas between the green and blue circles). Scattered are giant cells (black arrows).

The results from the mammogram and ultrasound supported the physical exam findings, prompting further CSD assessment with ultrasound-guided core biopsy. The biopsies showed necrotizing lymphadenitis that partially effaced the lymph node architecture. In the necrotic area, there were microabcesses with neutrophils and surrounding histiocytes, some of which were palisading (Figure 3). Giant cells were scattered throughout. Staining for cluster of differentiation 20 and cluster of differentiation 3 showed some residual follicles and interfollicular T cells. Other stains including acid-fast bacilli, methenamine silver stain, toxoplasmosis, and periodic acid-Schiff were all negative. Cat scratch stain was positive. To confirm the diagnosis, serology for B. henselae was subsequently ordered with the results of abnormal B. henselae IgG titer of 1:1024, and abnormal B. henselae antibody IgM titer of 1:32. 

Case 3

**Figure 4:**
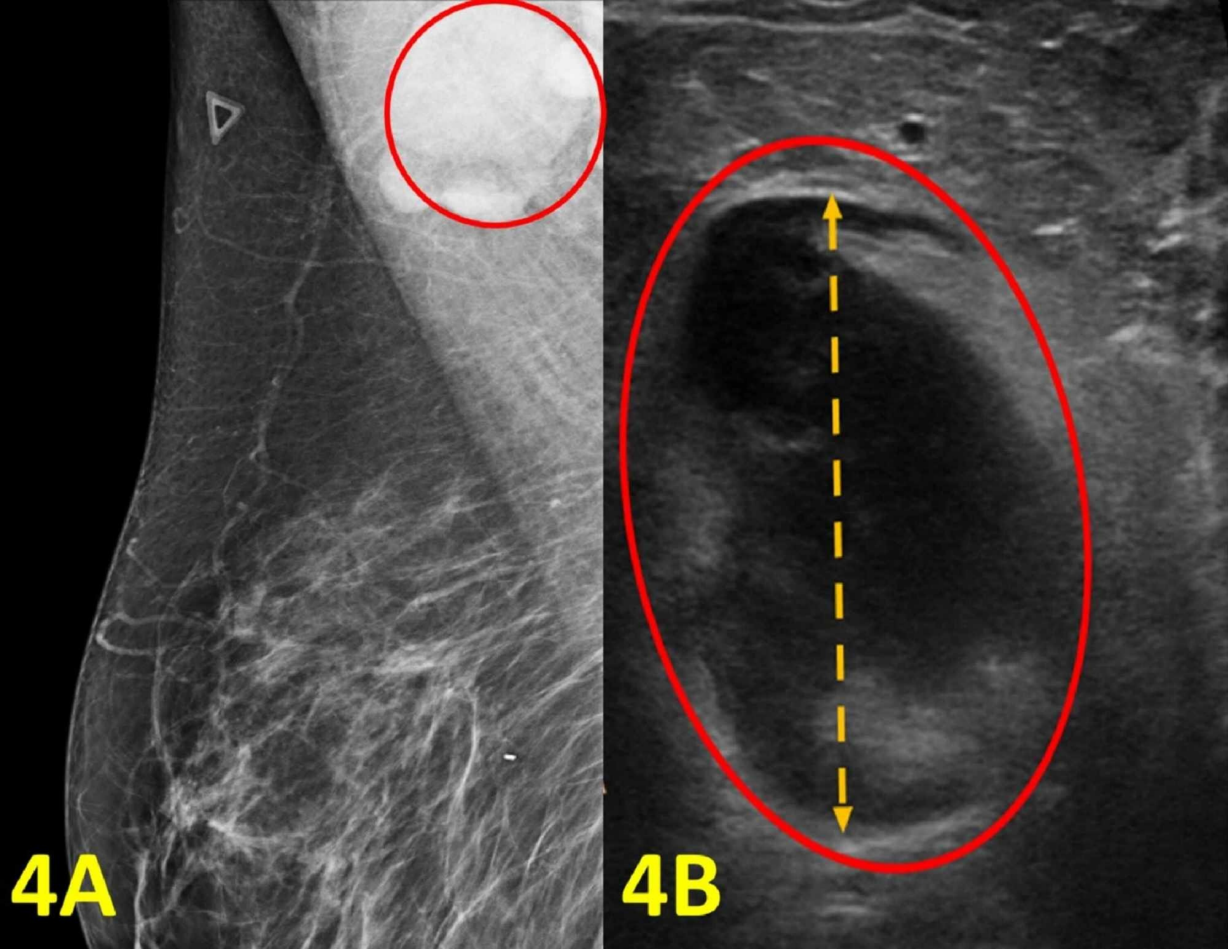
Imaging of the Right Breast and Right Axilla (A) Right breast diagnostic mammogram shows an enlarged lymph node in the right axilla (red circle). (B) Right axilla diagnostic ultrasound shows an enlarged axillary lymph node with cortical thickening (red oval) measuring 31 mm (orange double arrow).

A 49-year-old woman presented with arm pain for five days. The pain was located in the right armpit and sensitive to touch. The patient also reported bilateral ear pain. There were no chills, fevers, or night sweats. The patient had a normal screening mammogram five months prior. Her family history was significant for her mother with breast cancer and her father with gastric cancer. Socially, she had two cats. On physical examination, there was a painful mass below the right armpit with no overlying skin lesions. Because of the location of the mass and the family history of breast cancer, a repeat mammogram and an ultrasound were recommended. The imaging results were remarkable for a morphologically abnormal right axillary lymph node measuring 31 mm in diameter (Figure 4). Ultrasound-guided biopsy was performed, and the surgical pathology exam results were consistent with cat scratch lymphadenopathy. Flow cytometry revealed no detectable monoclonal B cells or immunophenotypically aberrant T cells, which diminished the concern for malignancy. Serology was not available for this case.

Case 4

**Figure 5:**
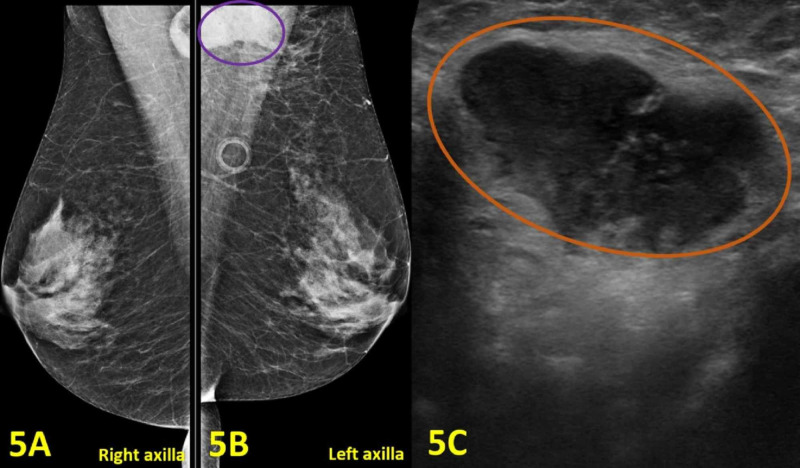
Imaging of Bilateral Breasts, Bilateral Axillae, and Left Axillary Lymphadenopathy (A) Mammogram of the right breast and axilla shows no abnormal findings. (B) Diagnostic mammogram of the left breast and axilla shows an enlarged lymph node with adjacent abscess measured 30 x 17 mm (purple oval). (C) Left axilla diagnostic ultrasound demonstrates an enlarged axillary lymph node with cortical thickening measuring 30 mm (orange oval).

A 58-year-old woman presented with breast and rib pain. She reported left axillary lymphadenopathy, abdominal fullness, and malaise. She denied fever and breast drainage. Her past medical history was significant for hepatitis C. Socially, she had two cats and was bitten by a cat a month prior. Review of systems was positive for fatigue and negative for appetite change, chills, diaphoresis, and fevers. Lung cancer screening CT, which was completed a day prior to this visit, showed an ill-defined mass measuring up to 3.5 cm in the left axilla. There was mild adjacent soft tissue stranding. These findings indicated an infectious, inflammatory, or malignant process. She was promptly treated with doxycycline for CSD, but also recommended to obtain a diagnostic mammogram and an ultrasound of the left axilla. The imaging showed an enlarged lymph node with an adjacent abscess, which altogether measured 30 x 17 mm in the left axilla (Figure 5).

Therefore, a lymph node biopsy and drainage of the abscess with culture were ordered. The lymph node biopsy revealed necrotizing granulomatous inflammation with the following differential diagnosis: CSD, tuberculosis, and other infectious entities. The abscess culture was negative for aerobic/anaerobic organisms and showed few polymorphonuclear leukocytes. Given her history of a cat scratch, symptoms, and pathology results, an infectious etiology was most likely. To confirm the diagnosis and rule out other causes, serology for B. henselae was ordered which showed high B. henselae IgG titer of >1:1024 and IgM titer of 1:128.

Case 5

**Figure 6:**
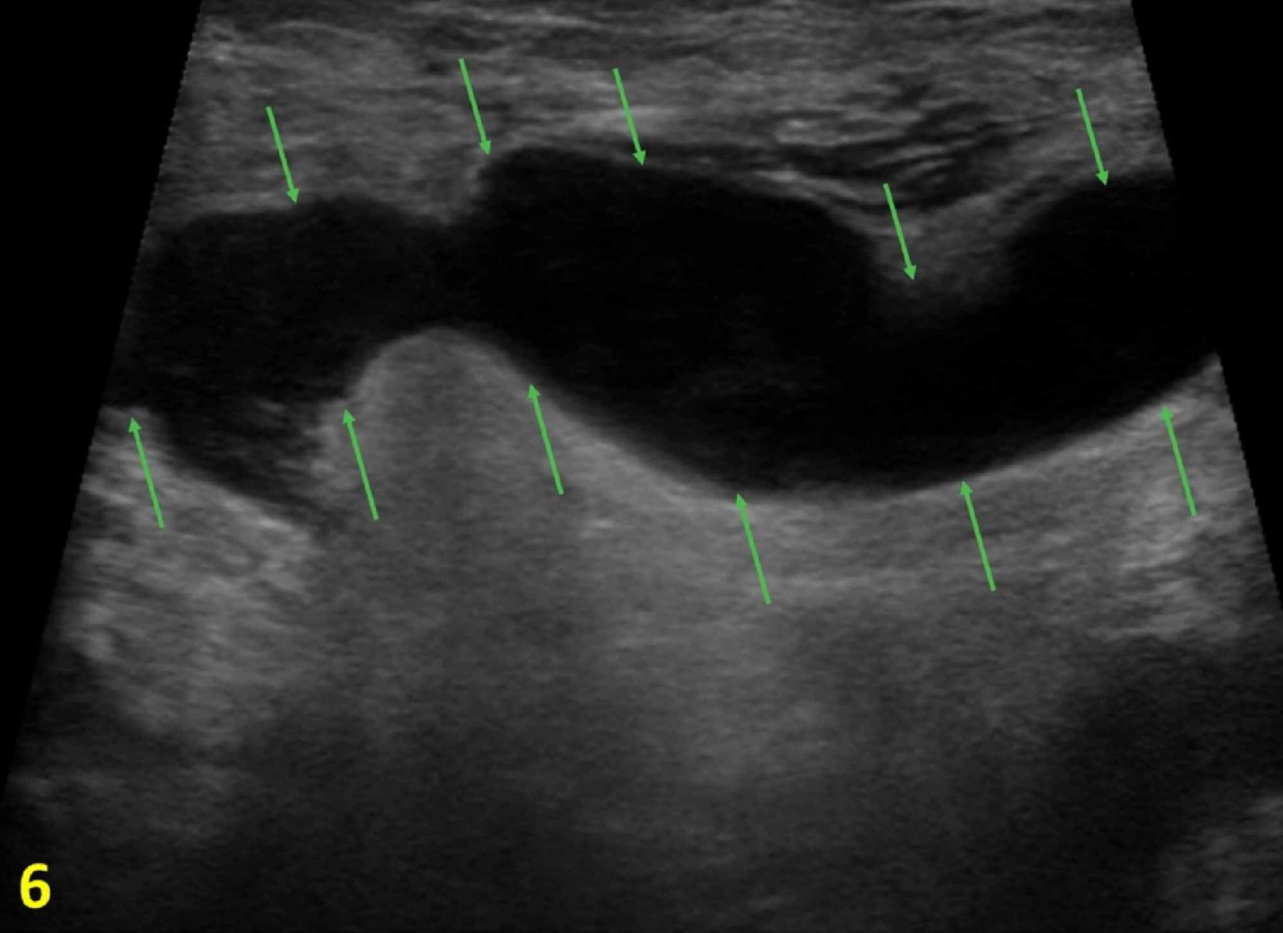
Right Axilla Diagnostic Ultrasound The image shows axillary lymphadenopathy with cortical thickening measuring 31 mm (outlined by green arrows).

A 25-year-old male patient with no significant past medical history presented with a three-day history of a tender mass in the right axilla along with fevers. The patient did not report any night sweats and weight loss. The mass showed no signs of drainage or infection. His physical examination revealed a 3 x 5 cm mobile, soft and non-nodular mass in the right axilla. An ultrasound of the right axilla showed a fluid-filled mass, which was concerning for an abscess. A needle aspiration was attempted without success; therefore, a referral to breast imaging clinic for a diagnostic ultrasound of the mass was initiated. Targeted ultrasound showed axillary lymphadenopathy at the site of the clinically palpable mass (Figure 6). Ultrasound-guided biopsy was performed, and pathology revealed suppurative granulomatous lymphadenitis suggestive of CSD. Serology was not available for this case.

## Discussion

The diagnosis of CSD is based on a combination of clinical, radiological, serological, and histological data. In terms of demographics, the selected cases featured young adults, representing the target population of CSD. In most of the cases, the patients had an identifiable source of cat exposure with or without constitutional symptoms along with subsequent axillary lymphadenopathy. Notably, a primary dermatological point of entry was identified distally from the area of axillary lymphadenopathy in case 1. Medical history including family history and personal history of carcinogenic exposure is important for including malignancy in the broad list of differential diagnoses. Cases 1, 2, and 3 had a positive family history of malignancy, warranting further diagnostic screening for breast cancer. Case 2 also had a personal history of smoking, which is associated with an increased risk of breast cancer [7]. Although men rarely develop breast cancer compared to women, it is imperative to encourage breast screening when warranted or when individuals have concerning symptoms and a positive family history of cancer, as in case 1.

CSD generally involves isolated axillary lymph nodes, sparing of the breast parenchyma. Neoplastic etiology will need to be ruled out as clinical findings of granulomatous lymphadenitis are generally non-specific with poorly defined enlarged axillary mass and soft tissue stranding on CT scan, as represented in case 4. In all of the presented cases, lymphadenopathy was the only defining feature on both mammogram and ultrasound. On ultrasound-guided biopsy, most of the cases of CSD feature necrotizing lymphadenitis. Recognition of the typical imaging findings along with a collection of the pertinent clinical history and relevant laboratory results will facilitate prompt investigation and lead to appropriate treatment of CSD.

The diagnosis of CSD is based on several of the following criteria: a history of cat exposure, primary cutaneous inoculation site, isolated regional lymphadenopathy, exclusion of other etiologies related to lymphadenopathy other than CSD, positive skin-test with specific antigen, and characteristic histopathological lymph node biopsy consistent with CSD [8]. Positive serological titer for B. henselae confirms the diagnosis of CSD. However, negative serology does not necessarily rule out CSD; therefore, suspicious cases of CSD should be treated empirically and presumptively. In the presented cases above, three patients have positive confirmatory antibody titers for B. henselae. Serologies were not available for case 3 and case 5. Although many cases of CSD are self-limiting, CSD is susceptible and responsive to several classes of antimicrobial agents, including ciprofloxacin, gentamicin, trimethoprim and sulfamethoxazole, clarithromycin, rifampin, and azithromycin [9]. Within the clinical setting, azithromycin is typically recommended as the first choice of antibiotic treatment.

## Conclusions

As evident by the five clinical presentations presented in this case series, the discovery of CSD is often reported in the breast imaging department during axillary lymph node assessment. Although isolated axillary lymphadenopathy is often benign, a metastatic process, if suspected, requires further imaging evaluation and lymph node biopsy. A detailed history, including cat exposure, identification of a primary inoculation site on physical exam, and clinical symptoms related to regional lymphadenitis can support a diagnosis of CSD and warrant empiric antibiotic therapy. On lymph node biopsy, CSD presents as necrotizing granulomatous lymphadenitis without any malignant cells. Furthermore, positive serology titer for B. henselae, an infectious bacterium of CSD, provides confirmation for CSD.

## References

[1] Walsh R, Kornguth PJ, Soo MS, Bentley R, DeLong DM (1997). Axillary lymph nodes: mammographic, pathologic, and clinical correlation. AJR Am J Roentgenol.

[2] Blanchard DK, Farley DR (2004). Retrospective study of women presenting with axillary metastases from occult breast carcinoma. World J Surg.

[3] Nelson CA, Saha S, Mead PS (2016). Cat-scratch disease in the United States, 2005-2013. Emerg Infect Dis.

[4] Illman JE, Terra SB, Clapp AJ, Hunt KN, Fazzio RT, Shah SS, Glazebrook KN (2018). Granulomatous diseases of the breast and axilla: radiological findings with pathological correlation. Insights Imaging.

[5] Povoski SP, Spigos DG, Marsh WL (2003). An unusual case of cat-scratch disease from Bartonella quintana mimicking inflammatory breast cancer in a 50-year-old woman. Breast J.

[6] Markaki S, Sotiropoulou M, Papaspirou P, Lazaris D (2003). Cat-scratch disease presenting as a solitary tumour in the breast: report of three cases. Eur J Obstet Gynecol Reprod Biol.

[7] Jones ME, Schoemaker MJ, Wright LB, Ashworth A, Swerdlow AJ (2017). Smoking and risk of breast cancer in the Generations Study cohort. Breast Cancer Res.

[8] Iannace C, Lo Conte D, Di Libero L (2013). Cat scratch disease presenting as breast cancer: a report of an unusual case. Case Rep Oncol Med.

[9] Conrad DA (2001). Treatment of cat-scratch disease. Curr Opin Pediatr.

